# CD38‐Targeted Theranostics of Lymphoma with ^89^Zr/^177^Lu‐Labeled Daratumumab

**DOI:** 10.1002/advs.202001879

**Published:** 2021-03-15

**Authors:** Lei Kang, Cuicui Li, Zachary T. Rosenkrans, Nan Huo, Zhao Chen, Emily B. Ehlerding, Yan Huo, Carolina A. Ferreira, Todd E. Barnhart, Jonathan W. Engle, Rongfu Wang, Dawei Jiang, Xiaojie Xu, Weibo Cai

**Affiliations:** ^1^ Department of Nuclear Medicine Peking University First Hospital Beijing 100034 China; ^2^ Departments of Radiology and Medical Physics University of Wisconsin – Madison Madison WI 53705 USA; ^3^ Department of Pharmaceutical Sciences University of Wisconsin – Madison Madison WI 53705 USA; ^4^ Department of Medical Molecular Biology Beijing Institute of Biotechnology Beijing 100850 China; ^5^ Department of Nuclear Medicine Union Hospital Tongji Medical College Huazhong University of Science and Technology Wuhan 430022 China

**Keywords:** CD38, daratumumab, Lu‐177, lymphoma, positron emission tomography (PET), radioimmunotherapy, theranostics

## Abstract

Lymphoma is a heterogeneous disease with varying clinical manifestations and outcomes. Many subtypes of lymphoma, such as Burkitt′s lymphoma and diffuse large B cell lymphoma, are highly aggressive with dismal prognosis even after conventional chemotherapy and radiotherapy. As such, exploring specific biomarkers for lymphoma is of high clinical significance. Herein, a potential marker, CD38, is investigated for differentiating lymphoma. A CD38‐targeting monoclonal antibody (mAb, daratumumab) is then radiolabeled with Zr‐89 and Lu‐177 for theranostic applications. As the diagnostic component, the Zr‐89‐labeled mAb is highly specific in delineating CD38‐positive lymphoma via positron emission tomography (PET) imaging, while the Lu‐177‐labeled mAb serves well as the therapeutic component to suppress tumor growth after a one‐time administration. These results strongly suggest that CD38 is a lymphoma‐specific marker and prove that ^89^Zr/^177^Lu‐labeled daratumumab facilitates immunoPET imaging and radioimmunotherapy of lymphoma in preclinical models. Further clinical evaluation and translation of this CD38‐targeted theranostics may be of significant help in lymphoma patient stratification and management.

## Graphical abstract

A CD38‐targeting monoclonal antibody (mAb, daratumumab) is radiolabeled with Zr‐89 and Lu‐177 for theranostics. As the diagnostic component, the Zr‐89‐labeled mAb is highly specific in delineating CD38‐positive lymphoma via positron emission tomography imaging, while the Lu‐177‐labeled mAb serves well as the therapeutic component to suppress tumor after a one‐time administration. Further clinical translation may be of high significance for lymphoma.

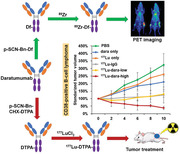

## 1 Introduction

Lymphoma is an umbrella term encompassing cancerous diseases stemming from lymphatic cells. As a heterogenetic cancer, many subtypes of lymphoma prove highly aggressive with dismal patient outcomes.^[^ ^1^ ^]^ Multiple myeloma (MM) is the second most common neoplastic hematologic disorder with ≈30 000 new cases annually in the United States.^[^ ^2^ ^]^ Although the application of immunotherapy and some proteasome inhibitors for MM has extended the median survival to more than 5 years, many patients inevitably develop drug resistance and become refractory.^[^ ^3^ ^]^ Considering the effectiveness of radiation therapy in MM since the malignant plasma cells outside the bone marrow are well documented to be radiosensitive,^[^ ^4^ ^]^ more specific diagnosis and radiation‐combined treatment may benefit MM patients with improved overall survival.

Monoclonal antibodies (mAbs) provide a powerful therapeutic agent in the era of precision medicine for cancer treatment. Along with the development of mAbs for cancer treatment, the identification of tumor‐specific biomarkers becomes a prerequisite for evaluation pre‐ or post‐treatment.^[^ ^5^ ^]^ Currently, almost all treatments or studies focused on CD20‐positive lymphomas using radiolabeled anti‐CD20 antibodies. However, some lymphoma cases do not express CD20 and would not benefit from these anti‐CD20 antibodies. CD38, a 45 kDa transmembrane glycoprotein receptor, is highly expressed on 95–100% of malignant plasma cells and at relatively low levels on normal cells,^[^ ^6^ ^]^ presenting a promising biomarker for several types of lymphomas, particularly MM. Moreover, CD38 has been generally recognized as a receptor and an ectoenzyme involved in the process of cytoplasmic calcium regulation and signal transduction, suggesting a potential therapeutic target for treatment.^[^ ^6^ ^]^ Thus, CD38 itself can play a role of theranostic in the area of lymphoma. Daratumumab (dara, from Janssen Biotech), a full human immunoglobulin G1 kappa mAb, is the first CD38 antibody approved by the Food and Drug Administration for the treatment of relapsed MM.^[^ ^6^, ^7^ ^]^ Dara can induce broad‐spectrum killing activity as a single agent with good tolerance, so it provides a good choice as a candidate for CD38‐targeted treatment.

Immuno‐positron emission tomography (immunoPET) combines the high sensitivity of PET imaging with the specificity of mAbs, offering a perfect way to assess the target expression noninvasively.^[^ ^8^ ^]^ Moreover, radioimmunotherapy (RIT) uses *α*‐, *β*‐, or Auger‐electron‐emitters to label mAbs for the treatment of cancer, offering a synergy of radiation and immunotherapy.^[^ ^9^, ^10^, ^11^ ^]^ As such, theranostic pairs based on mAbs have drawn significant attention in cancer management. The prospect of RIT in hematologic malignancies is better compared to solid malignancies since they are typically radiosensitive and multifocal.^[^ ^9^ ^]^ RIT can deliver systemic radiation to all lesion expressing specific biomarkers.^[^ ^12^ ^]^ As a clinical example, RIT has shown remarkable success in improving patient survival in relapsed/refractory B‐cell lymphoma.^[^ ^13^ ^] 131^I‐tositumomab (Bexxar) and ^90^Y‐ibritumomab (Zevalin), two radiolabeled anti‐CD20 mAbs, showed good response rates (47–68%) and favorable toxicity.^[^ ^14^, ^15^ ^]^ However, ^131^I is poorly retained in tumors and the iodine salts are rapidly cleared after degradation of the internalized mAbs.^[^ ^16^ ^]^ Additionally, ^90^Y is highly energetic and has a long emission path length (3.9 mm on average), which can induce unexpected damage to the surrounding normal tissue.^[^ ^17^ ^]^

To tackle the challenges, the theranostic role of a ^89^Zr/^177^Lu‐labeled CD38‐targeted mAb, daratumumab, is evaluated for immunoPET imaging and RIT of lymphoma. The positron emitter ^89^Zr has a physical half‐life of 78.4 h that matches the biological half‐life of intact mAbs in vivo, enabling ample time for tumor marker screening.^[^ ^18^, ^19^ ^]^ Lu‐177, on the other hand, emits short‐range *β*‐particles and *γ*‐photons with energies of 208 keV (11%) and 113 keV (6.4%), respectively.^[^ ^20^ ^]^ The decay half‐life of ^177^Lu (*t*_1/2_ = 6.65 d) is long enough to allow optimal tumor accumulation (5–10 d) for effective RIT.^[^ ^21^ ^]^ By comprehensively investigating the imaging, treatment, and toxicity of ^89^Zr/^177^Lu‐labeled daratumumab in lymphoma murine models, we aim to establish a promising theranostic role for early diagnosis and safe treatment of lymphoma in future clinic practices.

## 2 Results

### 2.1 Radiolabeling

**Figure 1:**
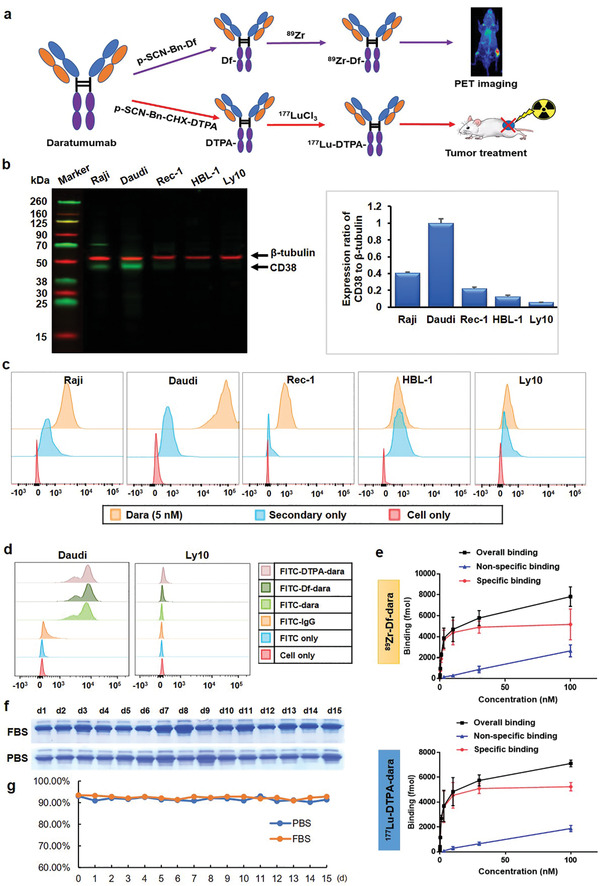
Theranostic design of radiolabeled daratumumab and in vitro evaluation. a) Scheme of the theranostic role of ^89^Zr‐ and ^177^Lu‐labeled daratumumab. b) Western blot showed that Daudi cells had the highest CD38 expression relative to *β*‐tubulin, whereas Ly10 cells had the lowest level. c) Flow cytometry proved the cellular binding affinity of daratumumab. d) Flow cytometry displayed that FITC‐labeled Df‐ or DTPA‐conjugated daratumumab had a similar cellular binding affinity. e) Receptor binding assay showed that ^89^Zr‐ and ^177^Lu‐labeled daratumumab had high binding ability in Daudi cells (*n* = 3). f) SDS‐PAGE showed that ^177^Lu–dara kept stability after being incubated in PBS and 10% FBS for 16 days. g) TLC presented that the radiochemical purities were higher than 90% over 16 days.

As outlined in **Figure** **1**a, ^89^Zr‐ and ^177^Lu‐labeled daratumumab were prepared by conjugating Df and DTPA to explore the potential theranostic application of daratumumab. After 1–2 h of incubation, labeling yields of more than 92% were achieved with the specific activities of 74–185 GBq g^−1^, as determined by TLC for both ^89^Zr and ^177^Lu‐labeled daratumumab. For imaging and therapy studies, similar labeling yields were consistently achieved.

### 2.2 Cellular CD38 Screening

Western blotting showed that Daudi cells had a significantly higher level of CD38 than Raji, Rec‐1, HBL‐1, and Ly10 cells, relative to *β*‐tubulin. Ly10 cells displayed the lowest CD38 expression (Figure 1b). Similar results found that daratumumab had a higher cell binding affinity for Daudi cells compared to the other cell lines using flow cytometry analysis (Figure 1c). Therefore, Daudi and Ly10 were used as a CD38 high‐ and low‐expressed cell line in this study, respectively.

### 2.3 Cellular Binding Affinity

The binding affinity of daratumumab was evaluated on Daudi and Ly10 cells. FITC‐labeled dara displayed similar levels of binding affinity after being conjugated with Df or DTPA. Dara‐related probes presented a strong shift to the right in the fluorescence histogram compared with control groups, suggesting that conjugation does not affect the binding ability (Figure 1d). For Daudi cell line, ^89^Zr–Df–dara was found to have a *B*_max_ value of 0.05 ± 0.00 fmol and a *K*_a_ value of (1.06 ± 0.16) × 10^−9^ m, whereas ^177^Lu–DTPA–dara had a similar *B*_max_ value (0.05 ± 0.00 fmol) and *K*_a_ value ((1.29 ± 0.39) × 10^−9^ m). Daudi cell had a high receptor density of (1.44 ± 0.21) × 10^5^ per cell (Figure 1e).

### 2.4 In Vitro Stability

After being incubated in PBS or 10% FBS, ^177^Lu–dara showed clear and uniform bands without degradation over 15 days, as revealed by SDS‐PAGE (Figure 1f). Besides, radio‐TLC results showed that the radiochemical purities were higher than 90% over 15 d, indicating excellent in vitro stability (Figure 1g).

### 2.5 ImmunoPET Imaging of ^89^Zr–Df–Dara

**Figure 2:**
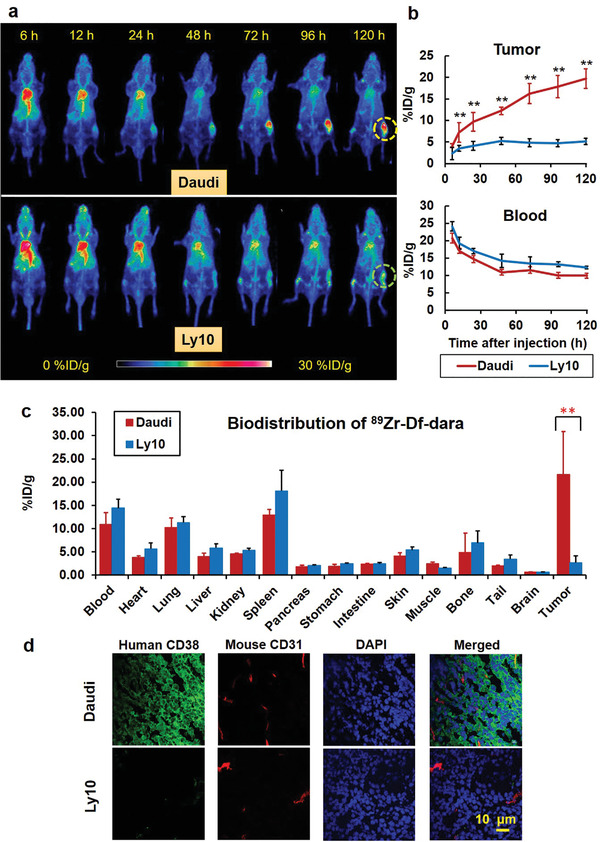
ImmunoPET imaging, biodistribution, and immunofluorescent staining results. a) The maximum intensity projection (MIP) PET images showed that ^89^Zr–dara exhibited an increasing and persistent tumor uptake in CD38‐positive Daudi models but near‐background uptake in CD38‐negative Ly10 models (*n* = 4). b) Quantitative data obtained via ROI analysis showed that the tumor uptake in Daudi models was significantly higher than in Ly10 models (*p* < 0.05). The blood uptake gradually decreased in both tumor models without significant difference. c) Ex vivo biodistributions at 120 h p.i. verified the imaging results. d) Immunofluorescent staining confirmed the strong intensity of CD38 signal in Daudi tumor tissue and limited signal in Ly10 tumor tissue.

Daudi and Ly10 tumor models were established for PET imaging with ^89^Zr–Df–dara. As shown in the maximum intensity projection (MIP) images, Daudi tumors exhibited an increased and persistent radioactive uptake at 5 days p.i. of ^89^Zr–Df–dara. By contrast, low levels of radioactive uptake were observed in Ly10 tumors (Figure **2**a). Quantitative data from ROI analysis (Figure 2b) showed that the tumor uptake increased from 4.2 ± 0.5 to 19.8 ± 2.3 %ID g^−1^ in Daudi models, whereas there was only a slight increase from 2.3 ± 0.7 to 5.1 ± 0.3 %ID g^−1^ in Ly10 models (*n* = 4) from 6 to 120 h. There were significant differences in tumor uptake between the two groups at all time points from 12 to 120 h p.i. (*p* < 0.05). The blood uptake decreased at all time points following the first scan but remained greater than 10 %ID g^−1^ in both tumor models. At the final time point of imaging (120 h p.i.), ex vivo biodistribution and immunofluorescent staining were performed. Daudi tumors displayed a significantly higher uptake of ^89^Zr–Df–dara with 21.6 ± 7.0 %ID g^−1^ compared to Ly10 tumors with 2.7 ± 1.2 %ID g−1 (*p* < 0.01). Other tissues showed no significant difference between the Daudi and Ly10 tumor models. Blood‐rich organs, such as lung and spleen, showed relatively high uptake (Figure 2c). Immunofluorescent staining verified the differential CD38 expression in Daudi and Ly10 tumors (Figure 2d). The resected Daudi tumor tissue displayed intense CD38 signals. By contrast, Ly10 tumors showed rare CD38 signals. Both tumor tissues expressed CD31, indicative of vascular growth in the tumor. Due to the prominent and prolonged tumor uptake of ^89^Zr–Df–dara in the Daudi tumor model, radioimmunotherapy using ^177^Lu‐labeled dara may be possible.

### 2.6 Tumor Size Monitoring after Radioimmunotherapy

**Figure 3:**
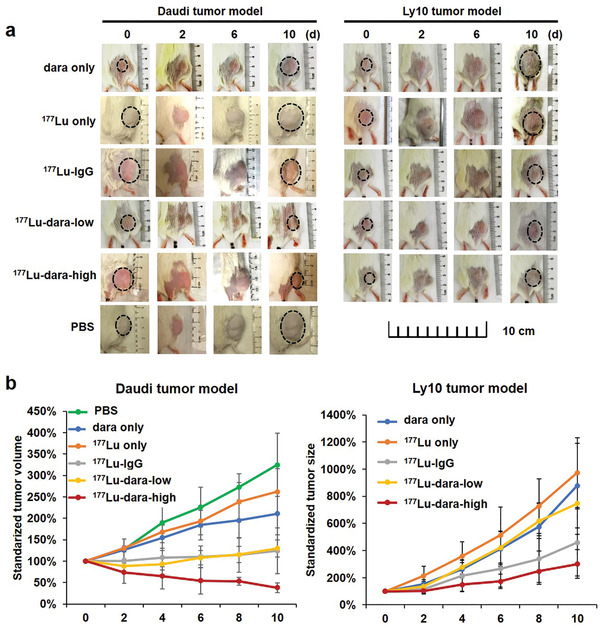
Tumor size monitoring. a) Representative tumor region photos were displayed for different groups in Daudi and Ly10 tumor models for 10 days. b) The standardized tumor volume for different groups in Daudi and Ly10 tumor models (*n* = 5–6).

Tumor sizes were monitored for 10 days after a single‐time injection of ^177^Lu–dara (high and low doses), ^177^Lu–IgG, ^177^Lu, dara only, and PBS in Daudi or Ly10 tumor models (*n* = 5–6, Figure **3** and Figures S2 and S3 (Supporting Information)). For Daudi models, ^177^Lu‐dara‐high (11.1 MBq) showed the strongest tumor suppression with standardized tumor volume at only 38.2 ± 11.4% at 10 days, significantly smaller than other treatment groups (PBS = 324.6 ± 73.8%, ^177^Lu = 261.5 ± 53.5%, ^177^Lu–IgG = 123.8 ± 54.5%, dara = 210.5 ± 49.1%; *p* = 0.00–0.04). ^177^Lu‐dara‐low (3.7 MBq) also showed tumor suppression with the standardized tumor volume at 130.0 ± 20.0%, significantly smaller than PBS, dara, and ^177^Lu control groups (*p* < 0.01). There was no significant difference between high and low dose treatments (*p* = 0.08). For Ly10 model, the standardized tumor volume of ^177^Lu‐dara‐high was 301.2 ± 104.6%, which was significantly smaller than that of dara (878.6 ± 311.5%), ^177^Lu (974.1 ± 256.5%), and ^177^Lu‐dara‐low groups (746.8 ± 227.7%) (*p* < 0.01), and no significant difference with ^177^Lu–IgG (458.2 ± 246.9%, *p* = 0.32). The Ly10 tumor volume of ^177^Lu‐dara‐low was not significantly smaller than dara and ^177^Lu groups (*p* = 0.83 and 0.20), suggesting its lack of tumor inhibition capacity. ^177^Lu and dara groups showed similar tumor volumes and displayed weak ability to inhibit tumor growth. These results showed that the therapeutic effect of ^177^Lu–dara is dose‐dependent and a higher dose has better treatment efficacy. Treatment using dara alone (50 µg) is not enough to inhibit tumor growth. Radioimmunotherapy plays a good role in the treatment of CD38‐positive tumor models.

### 2.7 ^18^F‐FDG Imaging Evaluation

**Figure 4:**
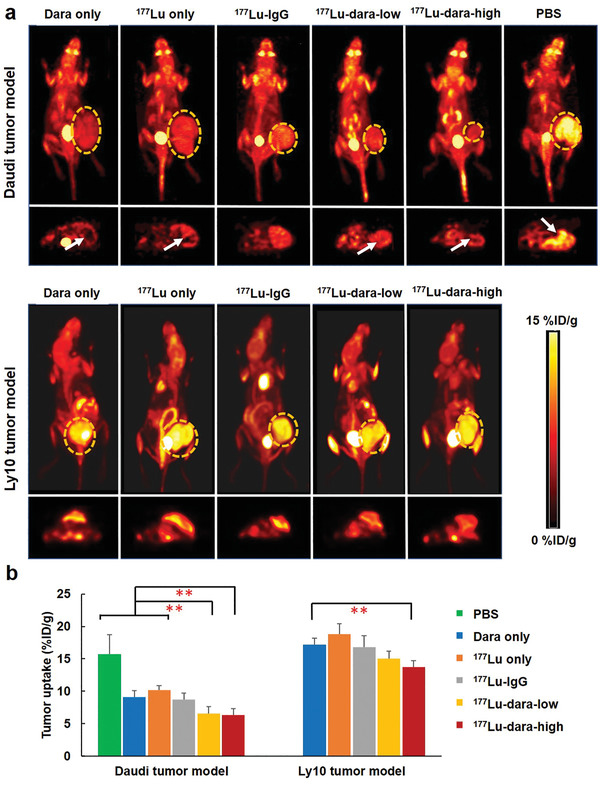
^18^F‐FDG PET imaging for evaluation of the therapeutic efficacy. a) Representative ^18^F‐FDG PET images were performed for different groups in Daudi and Ly10 models at 8 days (circles indicate tumor region, arrows point to the necrotic region). b) Quantitative data of tumor uptake were obtained after drawing region of interest on the tumor. ** represents *p* < 0.05 (*n* = 4).

Glucose metabolism in Daudi and Ly10 tumors was evaluated by ^18^F‐FDG PET imaging (Figure **4**a) and the maximum uptake value was used to assess treatment efficacy of different groups (Figure 4b). For Daudi models, the lowest ^18^F‐FDG uptake was found in ^177^Lu‐dara‐high group (6.3 ± 0.9 %ID g^−1^), which was significantly lower than PBS (15.7 ± 3.0 %ID g^−1^), dara (9.1 ± 1.0 %ID g^−1^), and ^177^Lu (10.2 ± 0.8 %ID g^−1^) groups (*p* = 0.00–0.04). There was no significant difference in FDG uptake between ^177^Lu‐dara‐high and ^177^Lu‐dara‐low (6.6 ± 1.1 %ID g^−1^) groups (*p* = 0.86). FDG uptake in dara group was only significantly lower than PBS group (*p* < 0.05). Additionally, the dark region with low uptake was observed in tumor treated with ^177^Lu–dara, indicating that small necrosis was found despite the small size of tumor. In comparison, the ^18^F‐FDG uptake in different groups of Ly10 models were all higher than 13 %ID g^−1^, with the lowest uptake value in ^177^Lu‐dara‐high group (13.7 ± 1.5 %ID g^−1^) and the highest value in ^177^Lu group (18.8 ± 1.6 %ID g^−1^). These results indicated that ^177^Lu‐dara‐high could effectively inhibit the glucose metabolism of CD38‐positive tumors.

### 2.8 Distribution of ^177^Lu‐Labeled Probes

**Figure 5:**
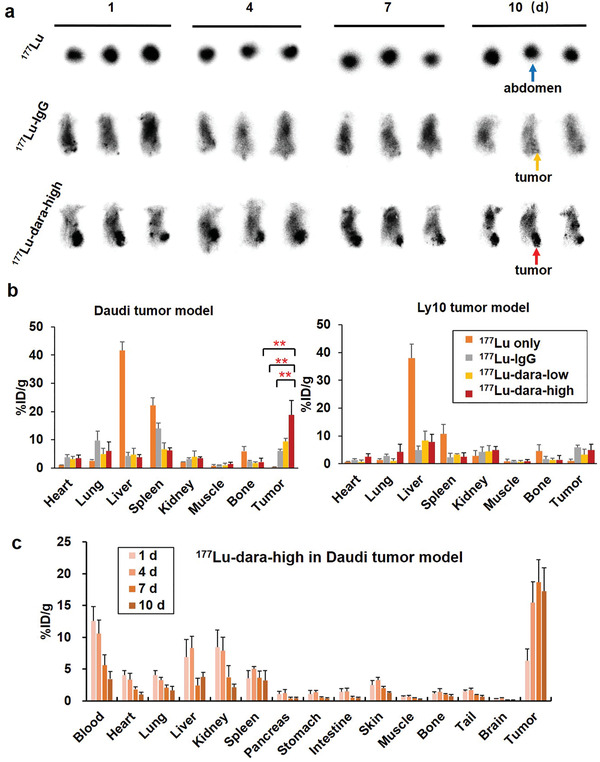
Verification of distribution of ^177^Lu‐labeled probes. a) Planar radiography was acquired for radioactive probes and showed significantly high tumor accumulation of ^177^Lu‐dara‐high, compared with ^177^Lu and ^177^Lu–IgG for Daudi model (*n* = 4). b) The biodistribution results at 10 days verified the high tumor uptake of ^177^Lu‐dara‐high in Daudi model, whereas no significant difference among four groups. ^177^Lu was mainly found in the liver and spleen, *n* = 4. c) The biodistribution of ^177^Lu‐dara‐high in Daudi model at 1, 4, 7, and 10 days showed a high and persistent tumor uptake up to 10 days p.i. of 17.2 ± 3.7 %ID g^−1^. Blood displayed a descendent uptake correlating with uptake in other normal organs.

Planar scintigraphy was used to evaluate the in vivo distribution of ^177^Lu‐labeled probes in Daudi and Ly10 tumor models (Figure S4, Supporting Information). Representative images of ^177^Lu‐dara‐high, ^177^Lu, and ^177^Lu–IgG in Daudi models were shown in Figure **5**a. Generally, all ^177^Lu‐labeled dara and IgG probes demonstrated diffuse radioactive distribution through the body in Daudi and Ly10 models, whereas ^177^Lu showed radioactive accumulation of only abdomen region. For Daudi models, tumor accumulation of ^177^Lu‐dara‐high was significantly higher than that of ^177^Lu–IgG, while ^177^Lu‐dara‐low showed scattered tumor accumulation. For Ly10 models, tumor regions could be hardly seen due to minimum tracer accumulation.

Ex vivo biodistribution results validated and quantified the planer imaging results (Figure 5b). Daudi tumor uptake of ^177^Lu–dara (18.9 ± 5.1 %ID g^−1^ for high dose and 9.4 ± 1.4 %ID g^−1^ for low dose) was significantly higher than that of ^177^Lu–IgG (6.0 ± 0.6 %ID g^−1^, *p* < 0.01). For comparison, Ly10 tumor uptake of ^177^Lu–dara (4.9 ± 2.2 %ID g^−1^ for high dose and 3.3 ± 2.0 %ID g^−1^ for low dose) was similar with ^177^Lu–IgG (5.9 ± 0.9 %ID g^−1^). Other blood‐rich organs, such as spleen, lung, and heart, presented relatively high uptake of ^177^Lu–dara and ^177^Lu–IgG. For ^177^Lu, the main uptake was found in liver for Daudi and Ly10 models (41.7 ± 3.0 and 37.9 ± 3.0 %ID g^−1^). Besides, the biodistribution of ^177^Lu‐dara‐high in Daudi models at different time points (Figure 5c) displayed high and persistent tumor uptake from 4 to 10 days p.i., with the peak uptake at 18.7 ± 3.6 %ID g^−1^ on 7 days. At the same time point, the tumor uptake of ^177^Lu–IgG and ^177^Lu was only 6.0 ± 0.6 and 0.3 ± 0.1 %ID g^−1^. The blood uptake decreased from 12.6 ± 2.3 to 3.5 ± 1.2 %ID g^−1^ over 10 days. Other major organs showed the same decreasing trend in tracer accumulation. These results confirmed that ^177^Lu–dara had high and specific binding with CD38‐positive tumors.

### 2.9 Histological Staining of Tumor Tissues

**Figure 6:**
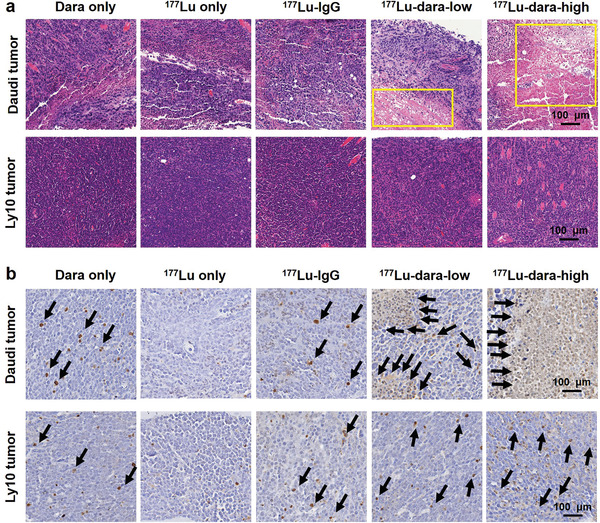
H&E and TUNEL staining of tumor tissues. a) H&E staining showed that the Daudi tumor tissues treated with ^177^Lu‐dara had necrosis inside (yellow box), whereas the Ly10 tumor tissues showed uniformly small lymphoma cells. b) TUNEL staining displayed that there was large scale of brown staining area inside the Daudi tumor treated with ^177^Lu–dara, suggesting apoptosis, but few staining in other groups.

H&E staining was performed to evaluate any morphological changes of tumor tissue and major organs including the liver, lung, kidney, heart, and small intestine. A large amount of small blue‐stained lymphoma cells could be seen around the Daudi and Ly10 tumor tissues. Necrosis was found in Daudi tumor tissues in both groups of ^177^Lu–dara and ^177^Lu–IgG, but more in the ^177^Lu‐dara‐high group. Ly10 tumor tissue showed no obvious change among different groups (Figure **6**a). In the ^177^Lu group, significant deformation of the liver structure and hepatic sinus was found, indicating liver toxicity caused by the accumulation of ^177^Lu. No necrosis or deformation was found in the other normal tissues including the lung, kidney, heart, and intestine, suggesting no significant toxicity in these tissues in different groups (Figure S5, Supporting Information).

Besides, TUNEL staining was used to display the apoptosis loci after the treatment. For Daudi models, the tumor tissue treated by ^177^Lu–dara showed the large scale of brown staining loci, indicating apoptosis with more loci in the high dose group. The tumors in the dara and ^177^Lu–IgG groups showed sporadic apoptosis loci. For Ly10 models, the tumors treated with ^177^Lu–dara showed a few apoptosis spots. Apoptosis was hardly found in the tumor in ^177^Lu group for both tumor models (Figure 6b). These results indicated the treatment efficacy of using ^177^Lu–dara in CD38‐positive tumors.

### 2.10 Body Weight Monitoring in CB/17 and BALB/c Mice

**Figure 7:**
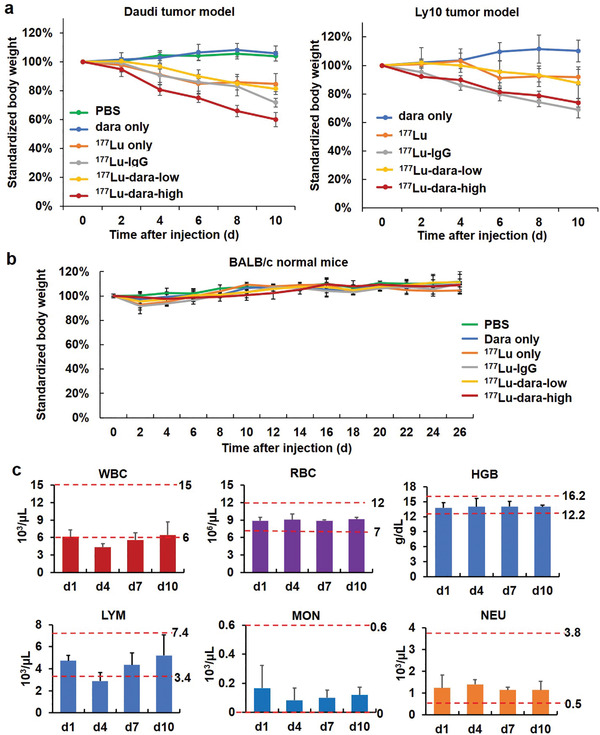
Biosafety evaluation. a) Standardized body weight of CB/17 SCID mice for Daudi and Ly10 tumor models were measured for 10 days and showed the decrease in all ^177^Lu‐related groups, suggesting potential toxicity (*n* = 4). b) The body weights of normal BALB/c mice were evaluated further after being treated similarly up to 26 d. The results showed no decrease or significant difference among different groups (*n* = 4, *p* > 0.05). c) Hematological analysis was performed for ^177^Lu‐dara‐high group in Daudi tumor model at 1, 4, 7, and 10 days. WBC, LYM, and MON cell counts dropped in the first 4 days but recovered gradually, indicating no sustained blood toxicity or inflammation after treatment.

The body weight was monitored for all groups in Daudi and Ly10 models to evaluate the mice's health conditions (Figure **7**a). For both Daudi and Ly10 models, a gradual drop of body weight was observed in all ^177^Lu‐related groups, especially ^177^Lu‐dara‐high and ^177^Lu–IgG, mainly due to the relatively high radiation dose received. The weight drop was a bit better in ^177^Lu and ^177^Lu‐dara‐low treatment groups. In the dara only and PBS groups, no weight drop was found. To prove whether the weight drop is related to the CB/17 mouse strain, BALB/c mice were further evaluated for their body weight changes following the same treatment for 26 days. As shown in Figure 7b, after an early drop at 2 days p.i., the body weight in all groups gradually increased. The standardized body weight after PBS and ^177^Lu‐dara‐high treatments at the final time point were 111.9 ± 2.0% and 109.1 ± 1.7%, respectively, without any significant differences (*p* > 0.05). These results indicated that the application of ^177^Lu–daratumumab in our study was safe in immunocompetent mouse models.

### 2.11 Hematological Results

Whole blood was collected and tested for major hematological parameters in Daudi models treated with ^177^Lu‐dara‐high (Figure 7c). WBC, LYM, and MON cell counts dropped within 4 days but recovered gradually, indicating no significant long‐term blood toxicity. No aberrant results were found in the WBC, LYM, MON, or NEU cell counts, suggesting no signs of inflammation after the treatment.

### 2.12 Dosimetry

Dosimetric extrapolation to an adult human male was calculated using the data of the biodistribution of ^177^Lu‐dara‐high and correlated with ^177^Lu decay data (Table S2, Supporting Information). The liver, lungs, and thymus were found with a relatively high dose, at around 0.03–0.04 mSv MBq^−1^. The estimated effective dose of total body is 0.15 ± 0.02 mSv MBq^−1^ (*n* = 4).

## 3 Discussion

The specific targeting ability of mAbs to tumor biomarkers provides an effectively theranostic approach for the selective diagnostic imaging of tumor cell antigens, evaluation of therapy efficacy, and delivery of ionizing radiation to the tumor after radiolabeling.^[^ ^22^ ^]^ Differences in the target expression and uptake of mAbs in tumor lesions affect the efficacy of mAb treatment.^[^ ^23^ ^]^ CD38 is overexpressed in some lymphomas and expressed at a relatively low level in normal tissues.^[^ ^19^ ^]^ Novel treatment strategies such as RIT are ideally suited to exploit these biomarkers, which is a therapeutic modality that combines immunotherapy and radiation therapy.^[^ ^24^ ^]^ In this study, the theranostic role of ^89^Zr/^177^Lu‐labeled daratumumab was evaluated in both CD38 high and low expressing tumor models. ^89^Zr–Df–dara could differentiate the CD38 expression levels in vivo, which is useful for the pre‐ or post‐CD38‐targeted therapy. ^177^Lu–dara could effectively suppress the growth of CD38‐positive tumors, whereas a higher dose was related to better treatment effect. Moreover, ^177^Lu–dara was found to have limited toxicity in the histological and hematological analysis. Therefore, this study offers an effective method for the theranostic of CD38‐positive cancer, particularly MM.

Lymphoma typically occurs in multiple lesions and is sensitive to radiation therapy.^[^ ^12^ ^]^ CD38 has been found to be highly expressed for some lymphoma such as MM.^[^ ^25^ ^]^ The first anti‐CD38‐humanized mAb daratumumab was subsequently approved for the treatment of relapsed MM. In our study, ^89^Zr‐labeled dara showed a high tumor uptake at around 20 %ID g^−1^ and low uptake in other nontarget organs for CD38‐positive models, providing excellent tumor contrast. It could also visualize the differential expression of CD38 noninvasively, thus is useful to evaluate the level of CD38 expression pre‐ or post‐therapy in suspected lesions. Besides, we found that ^177^Lu‐dara‐high reached its tumor uptake peak of 18.7 ± 3.6 %ID g^−1^ at 7 days and stayed high at 10 days (17.2 ± 3.7 %ID g^−1^) from biodistribution results, which provided a promising basis for the application of daratumumab‐based RIT. Based on the characteristics of gamma ray emission, ^177^Lu‐related groups could be visualized. ^177^Lu‐labeled dara showed high tumor uptake in Daudi models, even though the tumors kept shrinking over time. In comparison, ^177^Lu–IgG showed slight tumor uptake near body background, whereas ^177^Lu showed apparent abdominal uptake without any tumor uptake. Therefore, ^177^Lu–dara itself could behave as a theranostic agent for CD38‐targeted diseases.

The stability of labeled antibodies is a critical issue for RIT. In this study, we chose p‐SCN‐Bn‐CHX‐A″‐DTPA as the chelator due to the improved labeling efficiency (more than 90%) and simple direct labeling procedure. CHX‐A″‐DTPA‐conjugated antibodies showed less uptake in the blood pool and kidney, and reduced liver uptake than that of 1,4,7,10‐Tetraazacyclododecane‐1,4,7,10‐tetraacetic acid (DOTA).^[^ ^26^ ^]^ Moreover, the in vitro study of ^177^Lu–dara proved good stability with no degradation and off‐labeling after incubated in 10% FBS and PBS for up to 15 days, as shown by SDS‐PAGE and TLC analysis. CHX‐A″‐DTPA also provided good stability in vivo, which was deduced from the low bone uptake of less than 3 %ID g^−1^ for ^177^Lu–dara at 10 days in Daudi and Ly10 models. As shown by planar imaging and biodistribution results, ^177^LuCl_3_ group had high abdomen accumulation with the main distribution in the liver (41.7 ± 3.0 %ID g^−1^) and spleen (22.2 ± 2.8 %ID g^−1^). So, the low liver uptake of ^177^Lu–dara (3.7 ± 1.0 %ID g^−1^) also suggested its excellent in vivo stability, providing a good basis for therapy.

Although daratumumab has shown a robust therapeutic effect in the clinic, some patients cannot achieve a full response and acquire drug resistance later on.^[^ ^27^ ^]^ The primary advantage of RIT is that it combines the efficiency of mAbs and ionizing radiation. Additionally, radiation therapy can induce systemic antitumor immune reaction via the induction of immunogenic cell death, facilitation of antigen presentation or epitope, upregulation of surface receptors, and activation of T cells.^[^ ^24^, ^28^ ^]^ In MM, treatment efficacy of bortezomib and another anti‐CD38 murine mAb has been reported to be improved after radiolabeled with ^153^Sm or ^90^Y.^[^ ^4^, ^29^ ^]^ The addition of radiotherapy was hypothesized to increase therapeutic efficacy without introducing new chemotherapy drugs. In this study, less chemical amount of dara was used (50 µg per mouse) in each group than the recommended dose for clinical usage (16 mg kg^−1^, about 320 µg per mouse). Dara group showed limited tumor inhibitory effect with Daudi tumor size of 210.5 ± 49.1% at endpoint and significantly weaker than high dose of ^177^Lu–dara (38.2 ± 11.4%) or low dose of ^177^Lu–dara (130.0 ± 20.0%), although the same dose of dara was used in each group. Therefore, we proved that radiation therapy from ^177^Lu could play an effective role in this study.

Radiation dose is important for RIT. For the ^177^Lu‐related groups, we showed that the tumor inhibitory effect was correlated with the tumor radioactive distribution. For Daudi models, ^177^Lu‐dara‐high showed strong tumor suppress ability compared to three control groups: PBS, ^177^Lu, and ^177^Lu–IgG since it had a significantly higher tumor uptake. Although ^177^Lu‐dara‐low also had high tumor uptake, low total injected radioactive dose led to relatively low tumor radioactive, eliciting lower tumor inhibition than a higher dose. For Ly10 models, the standard tumor sizes were significantly larger than Daudi models. ^177^Lu‐dara‐high showed tumor inhibitory effect comparing to ^177^Lu, dara, and ^177^Lu‐dara‐low groups, it displayed a similar tumor inhibitory effect with ^177^Lu–IgG. Similar results were obtained that the high dose of 11.1 MBq could eradicate the tumor, whereas the lower doses of 3.7 and 7.4 MBq only caused a small delay of tumor growth and followed by regrowth.^[^ ^30^ ^]^ Considering the similar injected radioactive dose (11.1 MBq), this was due to the similar tumor uptake at about 5 %ID g^−1^ in Ly10 models. These findings highlighted that the effective therapy of ^177^Lu–dara was closely related to its radioactive dose and tumor accumulation. ^18^F‐FDG imaging is usually used to monitor the treatment efficacy in the clinic via presenting the glucose metabolism. Herein, we compared the FDG uptake of tumors in different groups. We found the FDG uptake in tumors treated by ^177^Lu–dara was significantly lower than other control groups, along with smaller tumor size. Necrosis could be displayed inside the ^177^Lu–dara‐treated tumor though the tumor size was not large, which was confirmed by H&E staining. Further, TUNEL showed large scale of apoptosis inside of the tumor. Taken together, these results suggested the efficient antitumor efficacy of ^177^Lu–dara by inhibiting the glucose metabolism and inducing apoptosis and necrosis.

In RIT, potential toxicity is what limits radiation doses and treatment side effects.^[^ ^31^ ^]^ Slow blood clearance of intact mAbs may increase radiation exposure in vivo. In our study, the body weights for both Ly10 and Daudi models in all ^177^Lu‐related groups were found to decrease, from about 16% to 40%, whereas dara and PBS groups did not show any decrease. Moreover, the body weight dropped without significant difference between ^177^Lu–dara and ^177^Lu–IgG with the same dose (11.1 MBq). Because CB/17 mice are hypersensitive to ionizing radiation,^[^ ^32^ ^]^ we thought that the decrease of body weight in all ^177^Lu‐related groups was related to this mice species. Thus, we evaluated the body weight of BALB/c mice after being treated similarly. The body weight increased with time for as long as 26 days. Moreover, H&E staining results showed no abnormal changes in major organs including liver, kidney, intestine, lungs, and heart. No abnormal blood cell counts were found after 10 days. In our previous study, an even higher dose of ^177^Lu‐labeled anti‐CD105 antibody showed no significant toxicity after measuring body weight and blood cell counts.^[^ ^26^ ^]^ A similar dose of ^90^Y‐labeled probes up to 9.25 MBq in the treatment of T‐cell lymphoma was proved to be safe with only transient mild cytopenia.^[^ ^33^ ^]^ Taken together, these results suggested ^177^Lu‐labeled dara would have limited toxicity and accepted biosafety if applied for immune efficiency subjects.

Based on the commercial availability and clinical application of daratumumab, radiolabeled daratumumab can provide a personalized approach that would be widely available for CD38‐positive lymphomas, especially MM. Several therapeutic *β*‐radionuclides have been considered for clinical RIT, such as ^131^I, ^90^Y, and ^177^Lu.^[^ ^34^ ^]^ The advantage of ^177^Lu is its theranostic role that emits *γ*‐rays for imaging and *β*‐rays with medium energy and proper tissue penetration of 1.5 mm.^[^ ^13^ ^]^ Its half‐life matches the pharmacokinetic characteristic of intact mAbs in vivo. Besides, some *α*‐particle‐emitted‐isotope‐labeled dara was evaluated for MM treatment, such as ^212^Pb and ^225^Ac.^[^ ^30^ ^]^ Even they had a theoretic advantage because of high linear‐energy transfer and shorter range with less potential toxicity, the relatively short half‐life and inconvenient supply of *α*‐particles limited their wide application. However, for the clinical application and translation, several issues such as an optimized dose, timing, radionuclide of choice, and administration times must be further evaluated. The promising result from this study suggests that this treatment strategy will allow targeted therapy and real‐time monitoring of response.

## 4 Conclusions

These studies found that CD38 served as a target for both imaging and therapy of lymphoma, meriting further exploration in the clinic. High and persistent tumor uptake and significant tumor inhibition were observed using radiolabeled daratumumab. Negligible toxicity of ^177^Lu–daratumumab was observed in the histological and hematological analysis. We believe that such an agent will show widely applicable clinical utility in many types of CD38‐positive hematological diseases. This study highlights the need for further investigation of the theranostic role of labeled daratumumab in future clinical applications.

## 5 Experimental Section

##### Radiolabeling

Dara was prepared for radiolabeling via the conjugation with SCN‐Bn‐deferoxamine (Df) or p‐SCN‐Bn‐CHX‐A″‐diethylenetriaminepentaacetic acid (DTPA, Macrocyclics) at the mole ratio of mAb:chelator at 1:5–1:10 following a standard protocol.^[^ ^19^, ^35^ ^]^ In brief, for ^89^Zr labeling, ^89^Zr‐oxalate in 4‐(2‐hydroxyethyl)‐1‐piperazineethanesulfonic acid (HEPES) buffer (0.5 m) was added into the Na_2_CO_3_ solution containing Df–dara (pH = 7.0) and mixed for 1 h at 37 °C. For ^177^Lu labeling, lutetium chloride (^177^LuCl_3_) in sodium acetate buffer (pH 6.5) was mixed gently with DTPA–dara (0.15 µg µCi^−1^) at 37 °C.^[^ ^35^ ^] 89^Zr‐oxalate was produced using a PETrace cyclotron (GE Healthcare). ^177^LuCl_3_ (*t*_1/2_ = 6.65 d) was purchased from PerkinElmer. Radiolabeled products were purified using PD‐10 columns (GE Healthcare) with a phosphate‐buffered saline (PBS) mobile phase. A radiolabeled nonspecific isotype control immunoglobulin G (IgG, Invitrogen) was prepared as a control group.

##### Cell Culture and Animal Models

Human B‐cell lymphoma cell lines, including Raji, Daudi, Rec‐1, HBL‐1, OCI‐LY10 (Ly10) were purchased from the American Type Culture Collection. All cells were cultured in RPMI 1640 medium with 10% fetal bovine serum (FBS) and 1% penicillin/streptomycin (Invitrogen) at 37 °C, 5% CO_2_.

All animal experiments were performed under protocols approved by the University of Wisconsin Institutional Animal Care and Use Committee and Peking University First Hospital Animal Committee (No. 201859). 4‐week‐old male CB17‐SCID immunodeficient mice (Envigo) were implanted with cells at the concentration of 5 × 10^6^ cells mL^−1^ to establish subcutaneous tumor models for treatment once the tumors reached a diameter of 5–10 mm. 4‐week‐old male BALB/c mice (Envigo) were used for safety and toxicity evaluation. Mice were monitored every other day for body weight and health conditions.

##### Western Blotting

Total proteins were extracted, electrophorized, and transferred to a membrane using a standard method.^[^ ^19^ ^]^ After being blocked with Odyssey blocking buffer (LI‐COR), the membrane was incubated with mouse anti‐human CD38 (1:1000) and rabbit anti‐human *β*‐tubulin (1:2000) antibodies (Novus Biologicals) overnight at 4 °C. Donkey anti‐mouse IRDye 800CW and goat anti‐rabbit IRDye 680RD antibodies (LI‐COR) were then used for secondary antibodies. Finally, the membrane was scanned and analyzed quantitatively using an Odyssey infrared imaging system (LI‐COR).

##### Flow Cytometry

Indirect and direct methods were used to test the cellular affinity according to previous protocols.^[^ ^19^ ^]^ Briefly, for indirect method, lymphoma cells were incubated with dara (5 × 10^−9^ m) and then a goat anti‐human secondary antibody (3 µg mL^−1^, ThermoFisher Scientific) in Flow Cytometry Staining Buffer Solution (eBioscience) at the concentration of 1 × 10^6^ cells mL^−1^. For direct method, N‐Hydroxysuccinimide –fluorescein (NHS–FITC, ThermoFisher)‐conjugated antibody was prepared and purified.^[^ ^25^ ^]^ Then, FITC‐conjugated DTPA– or Df–dara were tested and processed using a LSRFortessa cell analyzer (BD Biosciences) and FlowJo software (Tree Star) for the mean fluorescence intensities.

##### Cellular Binding Assay

The receptor binding affinity of ^89^Zr‐ and ^177^Lu‐labeled dara for Daudi cells was used to evaluate the immunoreactivities.^[^ ^36^ ^]^ Different concentrations of radiolabeled dara (0.03 × 10^−9^–100 × 10^−9^ m) were incubated with Daudi cells (1 × 10^6^ per well) at room temperature for 2 h and then counted with an automated gamma counter (PerkinElmer). The maximum binding ability (*B*_max_), affinity constant (*K*_a_), and receptor density of Daudi cells were calculated.

##### In Vitro Stability Evaluation

^177^Lu‐labeled dara (6.7 × 10^−6^ m) was incubated in PBS or 10% of FBS at room temperature for up to 15 days. Its stability was analyzed by sodium dodecyl sulfate polyacrylamide gel electrophoresis (SDS‐PAGE) and radio thin‐layer chromatography (TLC) analysis as previously reported.^[^ ^25^ ^]^

##### PET Imaging of ^89^Zr‐Labeled Antibodies

PET imaging was performed using an Inveon PET/CT scanner (Siemens) after intravenously injecting ^89^Zr‐labeled dara (≈7.4 MBq). At each time point, mice were anesthetized and scanned in a prone position. 20–30 million coincidence events were acquired for each mouse. Quantitative results were acquired by drawing regions of interest (ROI) at tumor and heart and analyzed using an Inveon Research Workspace (Siemens).

##### Therapeutic Administration and Monitoring

A single administration strategy was performed including both CD38‐positive and ‐negative tumor models and monitored for 10 days according to the previous study (scheme in Figure S1 in the Supporting Information).^[^ ^26^ ^]^ Two treatment groups at high doses (11.1 MBq) of ^177^Lu–DTPA–dara (^177^Lu‐dara‐high) and low dose (3.7 MBq) of ^177^Lu–DTPA–dara (^177^Lu‐dara‐low), and four control groups of ^177^Lu–DTPA–IgG (^177^Lu–IgG at 11.1 MBq), ^177^LuCl_3_ (^177^Lu at 11.1 MBq), dara, and PBS were analyzed (Table S1, Supporting Information). For single mouse, the total amount of dara or IgG injected was about 50 µg. After tail‐vein injection, each group (*n* = 5–6) was monitored every other day for tumor volume and body weight. Tumor volume was calculated by 1/2 × length × width^2^. The standardized tumor volume was calculated by dividing tumor volume at each time point by the initial tumor volume, multiplying by 100, and presented as %. The standardized body weight was measured and presented similarly. Humane endpoints were set as a body weight drop of more than 40%, or if the general health was too poor.

##### ^18^F‐Fluorodeoxyglucose (^18^F‐FDG) PET Imaging

^18^F‐FDG PET imaging was performed at 8 days postinjection (p.i.) to evaluate the therapeutic effect. The mice were kept fasting for at least 6 h before PET imaging. Each mouse was anesthetized for at least 50 min after injecting ^18^F‐FDG (≈7.4 MBq) until imaging. Twenty million coincidence events were acquired for each mouse. Quantitative analysis was also performed by drawing ROI on the tumor region.

##### Planar Scintigraphy

Planar scanning was acquired at 1, 4, 7, and 10 days after the injection of radioactive probes including ^177^Lu‐dara‐high, ^177^Lu‐dara‐low, ^177^Lu–IgG, and ^177^Lu only. Once anesthetized, mice were imaged at a supine position for 10 min using a Cyclone Plus Storage Phosphor System (PerkinElmer) or Discovery NM670 single‐photon‐emission computed tomography/computed tomography (SPECT/CT, GE Healthcare).

##### Biodistribution

Ex vivo biodistribution studies were performed for both ^89^Zr‐ and ^177^Lu‐labeled probes following the final time point in tumor‐bearing mice, as well as ^177^Lu‐dara‐high in Daudi tumor model at 1, 4, 7, and 10 days p.i. In brief, after mice were euthanized via CO_2_ asphyxiation, the organs of interest were harvested and wet‐weighed. Their radioactivities were measured using an auto gamma counter (PerkinElmer). The biodistribution results were presented as the percentage of injected dose g^−1^ (%ID g^−1^).

##### Hematological Analysis

Blood test was performed to evaluate any toxicity associate with treatment for ^177^Lu‐dara‐high group in Daudi tumor model at 1, 4, 7, and 10 d. Whole blood samples were collected after mice were euthanized and then analyzed using a VetScan HM5 hematology analyzer (Abaxis). The counts of white blood cell (WBC), red blood cell, hemoglobin, lymphocytes (LYM), monocytes (MON), and neutrophils (NEU) were tested from the blood sample.

##### Histological Staining

Tissue histological analysis included hematoxylin and eosin (H&E), immunofluorescence, and terminal deoxynucleotidyl transferase deoxyuridine triphosphate nick end labeling (TUNEL) staining. H&E staining was performed on tumor, heart, lung, liver, kidney, and small intestine tissues following standard protocols.^[^ ^37^ ^]^ Immunofluorescent staining was performed on Daudi and Ly10 tumor tissues to show the expression of CD38 and blood vessels. DyLight550‐conjugated anti‐CD38 antibodies and AlexaFluor488‐conjugated anti‐CD31 (Novus Biologicals) were used as antibodies. Horseradish peroxidase (HRP) ‐ 3,3‐diaminobenzidine (DAB) TUNEL assay kit (Abcam, No. ab206386) was used to test the apoptosis of tumor tissues, as well as some major organs in the group of ^177^Lu‐dara‐high, following the standard protocol. The brown color represented apoptosis loci. Morphology and staining were observed and recorded using an A1R microscope (Nikon).

##### Radiation Dosimetry Prediction

The radiation absorbed doses were calculated from the biodistribution data of ^177^Lu‐dara‐high in Daudi models. A conservative estimate of dose to a tumor was calculated using dose‐to‐sphere modeling of OLINDA/EXM provided by the OLINDA/EXM software.^[^ ^38^ ^]^

##### Statistical Analysis

All quantitative data were presented as mean ± standard deviation. Repeated‐measure analysis of variance (ANOVA) was used for comparisons among groups by SPSS (v20.0). *p* values less than 0.05 were considered statistically significant.

## Conflict of Interest

Weibo Cai is scientific advisor, stockholder, and grantee of Focus‐X Therapeutics, Inc. The other authors declare no conflict of interest.

## Supporting information

Supporting Information

## Data Availability

Research data are not shared.
