# Supplementary material for: CD38‐Targeted Theranostics of Lymphoma with 89Zr/177Lu‐Labeled Daratumumab
Source: Adv Sci (Weinh). 2021 Mar 15;8(10):2001879. doi: 10.1002/advs.202001879 (PMC8132161; doi:10.1002/advs.202001879)
Supplement: Supplementary file 1 — Supporting Information [file ADVS-8-2001879-s001.pdf]

© 2021 Wiley-VCH GmbH

ADVANCED SCIENCE Open Access

Supporting Information

for *Adv. Sci.*, DOI: 10.1002/advs.202001879

CD38-targeted theranostics of lymphoma with 89Zr/177Lu-labeled daratumumab

Lei Kang, Cuicui Li, Zachary T. Rosenkrans, Nan Huo, Zhao Chen, Emily B. Ehlerding, Yan Huo, Carolina A. Ferreira, Todd E. Barnhart, Jonathan W. Engle, Rongfu Wang, Dawei Jiang,* Xiaojie Xu,* and Weibo Cai*

Table S1. Information about different therapeutic study groups.

Group number	Study group	Tumor	Number (n)	Radioactivity (MBq)	Treatment
1	Daratumumab only	Daudi	5	none	50 µg of daratumumab in 1× PBS
2		LY-10	5		
3	^{177}Lu only	Daudi	5	11.1	11.1 MBq of $^{177}LuCl_3$ in 1× PBS
4		LY-10	5		
5	^{177}Lu-DTPA-IgG	Daudi	5	11.1	11.1 MBq of ^{177}Lu-DTPA-IgG in 1× PBS (50 µg of IgG total)
6		LY-10	5		
7	^{177}Lu-DTPA-daratumumab low dose	Daudi	6	3.7	3.7 MBq of ^{177}Lu-DTPA-daratumumab + 30 µg of daratumumab in 1× PBS (50 µg of daratumumab total)
8		LY-10	6		
9	^{177}Lu-DTPA-daratumumab high dose	Daudi	6	11.1	11.1 MBq of ^{177}Lu-DTPA-daratumumab in 1× PBS (50 µg of daratumumab total)
10		LY-10	6		
11	PBS	Daudi	5	None	1× PBS

Table S2. Human dosimetry prediction using the biodistribution data of ^{177}Lu-dara-high and the decay data of ^{177}Lu in Daudi model (n = 4).

	**Radiation absorbed dose**			
	mGy/MBq	S.D.	RAD/mCi	S.D.
**Adrenals**	1.21E-04	2.26E-05	4.49E-04	8.35E-05
**Brain**	6.18E-04	1.61E-04	2.29E-03	5.97E-04
**Breasts**	2.09E-03	3.63E-04	7.72E-03	1.34E-03
**LLI Wall**	3.81E-03	7.39E-04	1.41E-02	2.73E-03
**Small Intestine**	2.51E-04	3.53E-05	9.30E-04	1.31E-04

**Stomach Wall**	9.77E-03	1.81E-03	3.61E-02	6.71E-03
**ULI Wall**	8.84E-05	1.70E-05	3.27E-04	6.30E-05
**Kidneys**	5.96E-03	6.33E-03	2.21E-02	2.34E-02
**Liver**	3.04E-02	7.66E-03	1.12E-01	2.84E-02
**Lungs**	4.15E-02	9.27E-03	1.53E-01	3.43E-02
**Muscle**	1.47E-04	2.89E-05	5.44E-04	1.07E-04
**Ovaries**	6.49E-03	1.25E-03	2.40E-02	4.61E-03
**Pancreas**	3.49E-04	1.04E-04	1.29E-03	3.83E-04
**Red Marrow**	3.40E-03	6.37E-04	1.26E-02	2.36E-03
**Osteogenic Cells**	1.03E-03	1.97E-04	3.81E-03	7.30E-04
**Skin**	3.03E-04	5.84E-05	1.12E-03	2.16E-04
**Spleen**	1.21E-03	2.74E-04	4.49E-03	1.01E-03
**Thymus**	3.77E-02	3.23E-02	1.39E-01	1.20E-01
**Thyroid**	1.62E-03	3.06E-04	6.01E-03	1.13E-03
**Urinary Bladder Wall**	1.56E-03	3.02E-04	5.77E-03	1.12E-03
**Uterus**	8.05E-05	1.55E-05	2.98E-04	5.74E-05
**Total Body (mSv/MBq)**	1.48E-01	2.18E-02	5.48E-01	8.06E-02

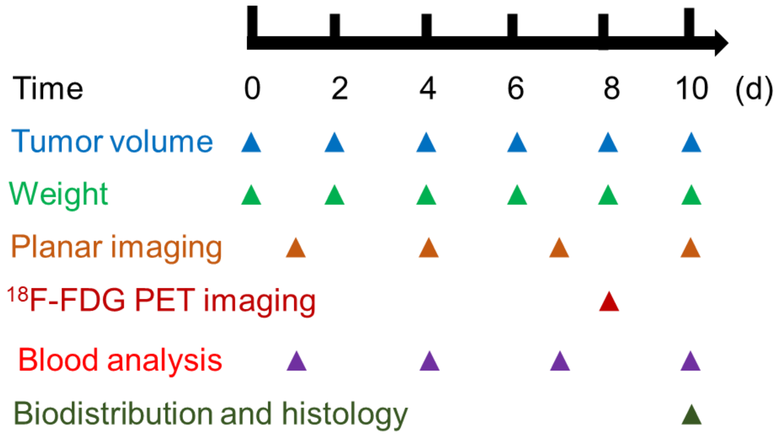

Figure S1 Scheme of the timeline after the treatment

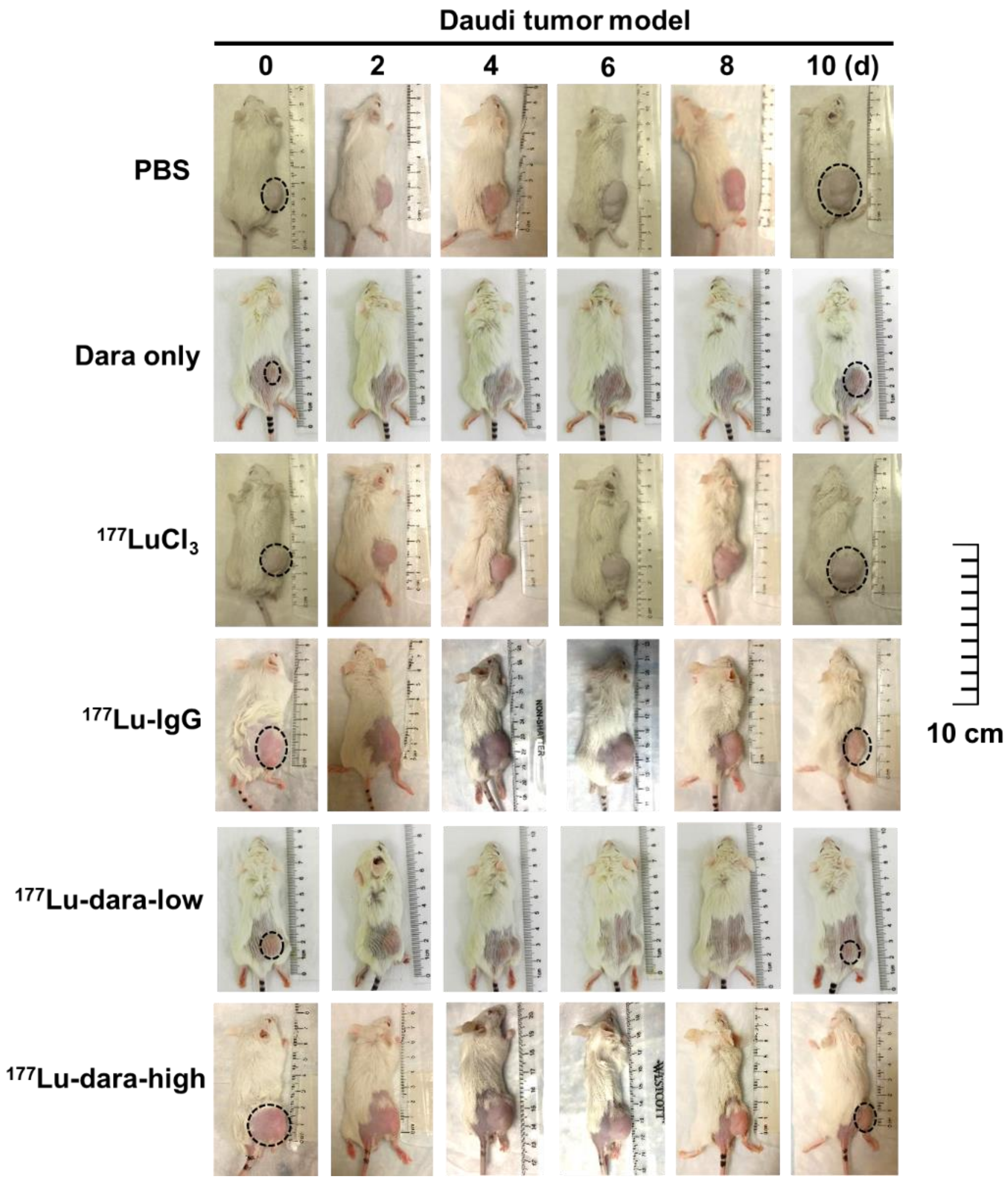

Figure S2 Representative photos of CB/17 mice in Daudi model treated with ^{177}Lu-dara-high or -low, ^{177}Lu-IgG, ^{177}Lu, dara, and PBS. The size of tumors was the smallest in the ^{177}Lu-dara-high group (32.8 ± 13.8% of initial at 10 d and was the largest in the PBS group (276.7 ± 0.6%).

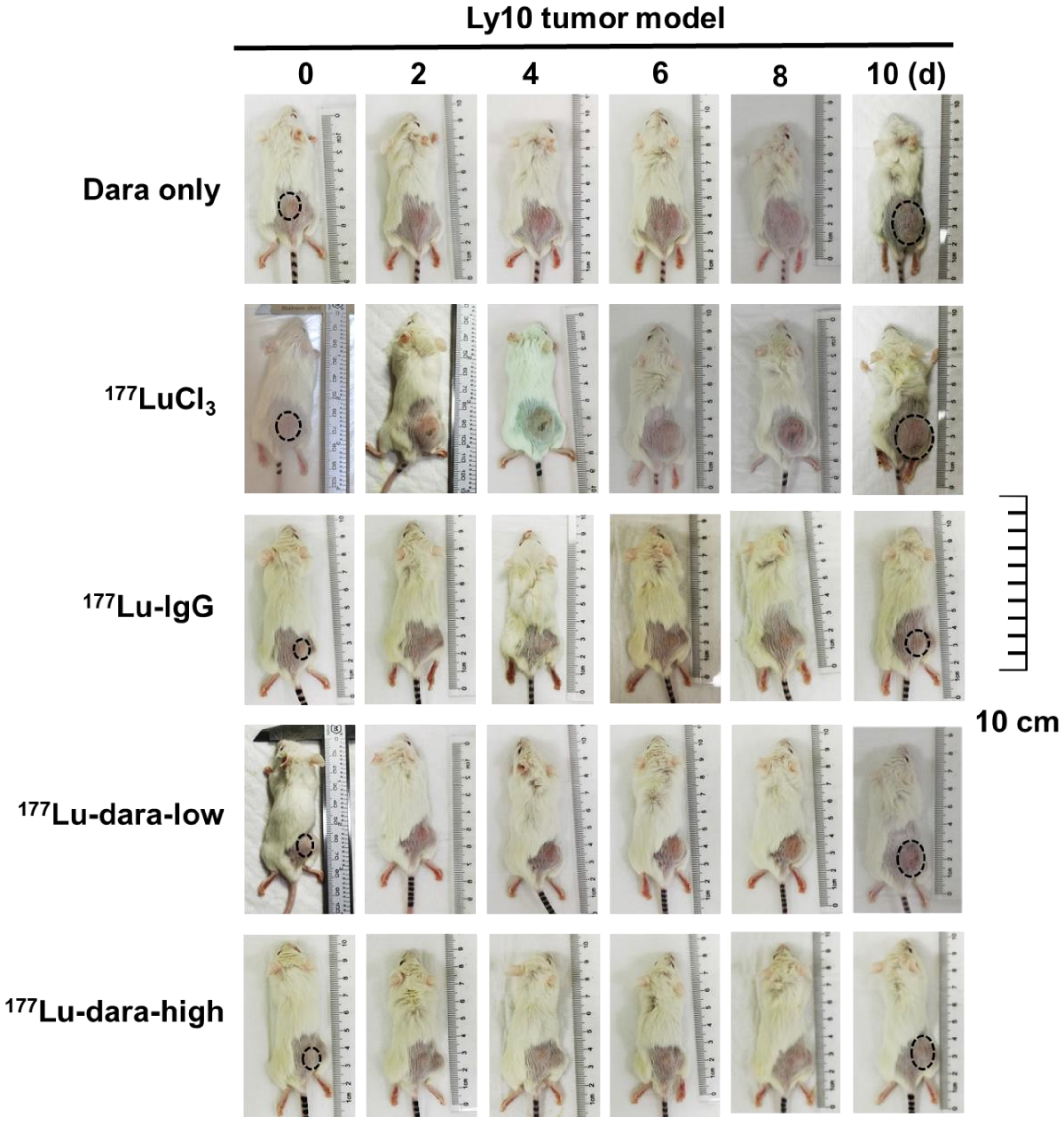

Figure S3 Representative photos of CB/17 mice in Ly10 model treated with ^{177}Lu-dara-high or -low, ^{177}Lu-IgG, ^{177}Lu, and dara. The tumor size increased with time in different groups, from 301.2 ± 104.6 % to 878.6 ± 311.5 %.

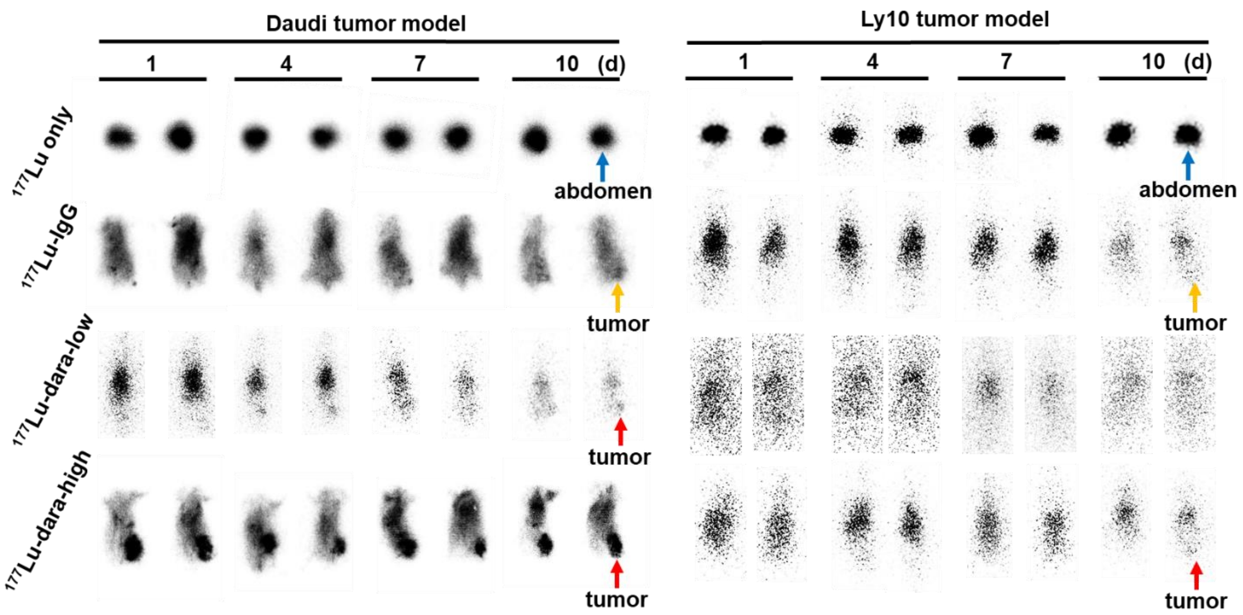

Figure S4 Planar radiography of Daudi and Ly10 tumor models after injection of different ^{177}Lu-related probes. High tumor accumulation could be seen in ^{177}Lu-dara-high group, whereas no clear tumor images could be displayed in other groups for both Daudi and Ly10 models. Slight tumor uptake could be seen for ^{177}Lu-dara-low group in Daudi model. In ^{177}Lu groups, obvious abdomen accumulation was shown for two tumor models.

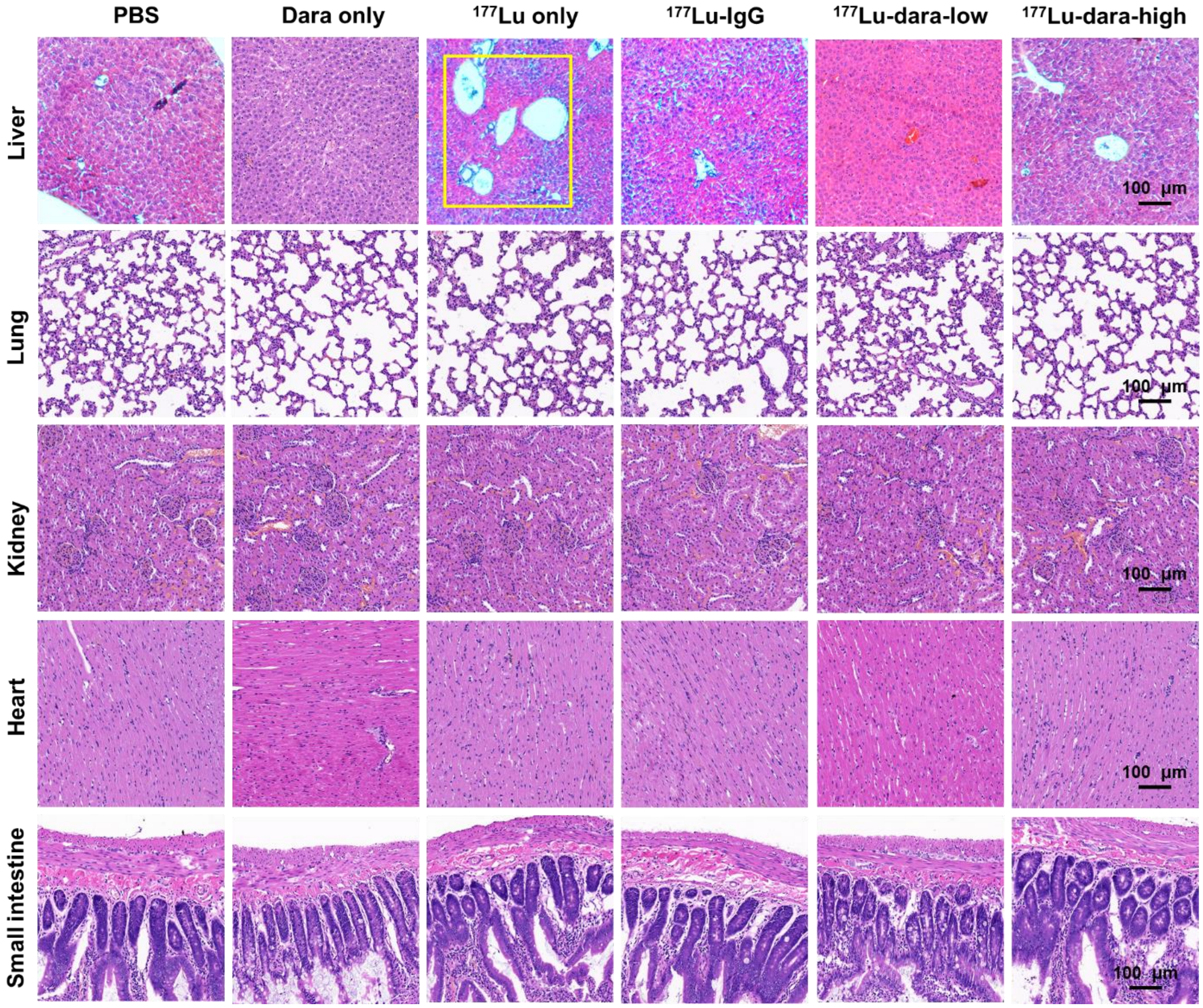

Figure S5 H&E staining of major organ tissues in Daudi tumor model. For ^{177}Lu group, the liver was found with significant deformation of liver structure and hepatic sinus (yellow box). No necrosis or deformation was found in other tissues, suggesting no significant toxicity in these tissues for all the groups.

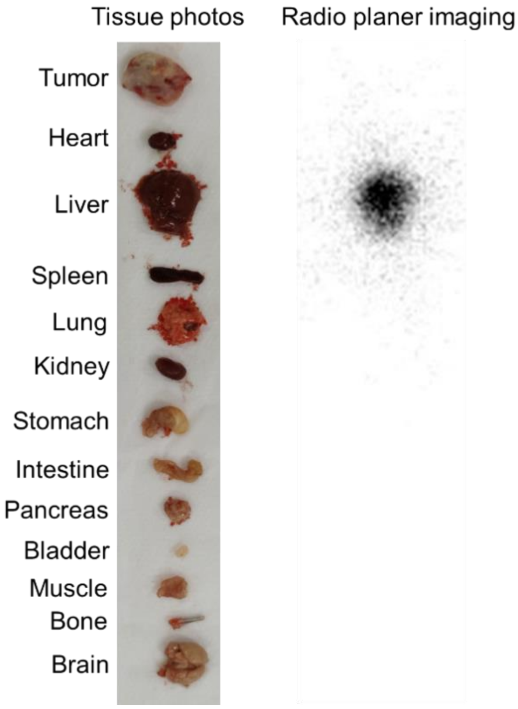

Figure S6 Ex vivo planar scintigraphy of Daudi bearing mouse for the ^{177}Lu group at 10 d.

The liver showed prominent radioactive accumulation among all the removed tissues.
